# Optimization of Adhesion Strength and Microstructure Properties by Using Response Surface Methodology in Enhancing the Rice Husk Ash-Based Geopolymer Composite Coating

**DOI:** 10.3390/polym12112709

**Published:** 2020-11-16

**Authors:** Mohd Salahuddin Mohd Basri, Faizal Mustapha, Norkhairunnisa Mazlan, Mohd Ridzwan Ishak

**Affiliations:** 1 Department of Process and Food Engineering, Faculty of Engineering, Universiti Putra Malaysia, UPM Serdang 43400, Selangor, Malaysia; 2 Laboratory of Halal Science Research, Halal Products Research Institute, Universiti Putra Malaysia, UPM Serdang 43400, Selangor, Malaysia; 3 Department of Aerospace Engineering, Faculty of Engineering, Universiti Putra Malaysia, UPM Serdang 43400, Selangor, Malaysia; faizalms@upm.edu.my (F.M.); norkhairunnisa@upm.edu.my (N.M.); mohdridzwan@upm.edu.my (M.R.I.); 4 Institute of Advanced Technology (ITMA), Universiti Putra Malaysia, UPM Serdang 43400, Selangor, Malaysia; 5 Institute of Tropical Forestry and Forest Products (INTROP), Universiti Putra Malaysia, UPM Serdang 43400, Selangor, Malaysia

**Keywords:** geopolymer, coating, optimization, polymer, adhesion

## Abstract

As a result of their significant importance and applications in vast areas, including oil and gas, building construction, offshore structures, ships, and bridges, coating materials are regularly exposed to harsh environments which leads to coating delamination. Therefore, optimum interfacial bonding between coating and substrate, and the reason behind excellent adhesion strength is of utmost importance. However, the majority of studies on polymer coatings have used a one-factor-at-a-time (OFAT) approach. The main objective of this study was to implement statistical analysis in optimizing the factors to provide the optimum adhesion strength and to study the microstructure of a rice husk ash (RHA)-based geopolymer composite coating (GCC). Response surface methodology was used to design experiments and perform analyses. RHA/alkali activated (AA) ratio and curing temperature were chosen as factors. Adhesion tests were carried out using an Elcometer and a scanning electron microscope was used to observe the microstructure. Results showed that an optimum adhesion strength of 4.7 MPa could be achieved with the combination of RHA/AA ratio of 0.25 and curing temperature at 75 °C. The microstructure analysis revealed that coating with high adhesion strength had good interfacial bonding with the substrate. This coating had good wetting ability in which the coating penetrated the valleys of the profiles, thus wetting the entire substrate surface. A large portion of dense gel matrix also contributed to the high adhesion strength. Conversely, a large quantity of unreacted or partially reacted particles may result in low adhesion strength.

## 1. Introduction

In many applications, such as oil and gas, building construction, offshore structures, ships and bridges, steel is widely used as the main component. In order to provide effective protection against corrosion and improve the biological performance of the substrate, surface coating technology is applied [1]. The performance and reliability of coatings rely on the adhesion between the coating and the substrate surface. In addition, expansion and contraction during temperature changes need to be considered in order for the coating material to perform over an extended period of time. Therefore, the coating material itself must be durable and have good interfacial bonding between coating and substrate.

Without excellent adhesion strength between coating and substrate, the component underneath the coating will be easily exposed to harsh environments such as corrosive fluids, flame and heat, and abrasive particles when the coating delaminates or breaks off into small flakes or pieces. Once exposed and unprotected, the component will not function properly or fail prematurely. According to Hull, et al. [2], there are four different mechanisms of coating adhesion, namely interfacial adhesion, inter-diffusion adhesion, intermediate layer adhesion, and mechanical interlocking adhesion. Based on the conclusions, two out of the four mechanisms provide stronger adhesion bonding, i.e., (i) inter-diffusion adhesion, in which the coating is mutually dispersed into the substrate, and (ii) mechanical interlocking, in which a crack propagation barrier is provided by a rough interface between coating and substrate. Numerous studies were conducted on the preparation of fly ash-based and metakaolin-based geopolymer composite coating (GCC) on metal substrate using the dip coating technique. However, there are no reports in the literature that deal with the use of RHA as the only aluminosilicate source in the production of geopolymer coatings.

Geopolymers are attractive materials for scientists globally for their excellent properties, including high compressive strength, low shrinkage and resistance to fire and acid [3,4,5]. Studies on numerous geopolymer types, including geopolymer concrete [6], precast paving cement-free brick [7] and lightweight geopolymer composites [8] have been conducted. As an inorganic polymer which is based on synthetic aluminosilicate materials [9], geopolymers have potential in coating applications as surface protectors and insulators for various surfaces, including metal. Pozzolanic wastes such as fly ash, metakaolin and blast furnace slag are commonly used to form GCC and coated either on ceramic or metal substrates [10]. However, to the best of our knowledge, there is no documentation as yet on the implementation of an optimization process to achieve the best GCC composition. Rice husk ash is used in this research as the main component in the development of GCC, and response surface methodology (RSM) as an optimization tool to identify the most suitable coating composition. This method will also be used to validate the predicted optimal value.

Temuujin, et al. [11] studied the adhesion strength of metakaolin-based and fly ash-based geopolymers coating on stainless and mild steel plates. Adhesion tests were conducted in accordance with the ASTM D4541 standard (ASTM D4541). The substrates were coated using a dip coating technique and the coated samples were cured at 70 °C for 24 h. For coating on stainless and mild steel plates, the adhesion strength for samples with a Si/Al ratio of 2.5 for both substrates was above 3.5 MPa [10]. There were no results for geopolymer coatings with a Si/Al ratio of 1 and 2 due to the observed weak adhesion bonding between coating and substrate. The authors concluded that the bonding was likely to be due to physical adhesion rather than chemical due to the absence of bond formation between Si and Fe atoms.

Similar tests have been conducted by Yusof, et al. [12], with a geopolymer coating thickness of 0.2 mm to 0.5 mm and the adhesion strength was found to be above 3.5 MPa. Temuujin, Minjigmaa, Rickard, Lee, Williams and Van Riessen [11], also conducted an adhesion test on the FA-based geopolymer coating using mild and stainless steel as the substrates. With a coating thickness of 0.5 mm, the adhesion strength was found to be above 3.5 MPa for geopolymer coated on mild steel and in the range of 1.2 MPa to 1.4 MPa for geopolymer coated on stainless steel. The high adhesion strength of geopolymer corresponded to a Si/Al ratio of 3.5 while the low adhesion was in the range of 1.2 MPa to 1.4 MPa, with a Si/Al ratio below 3.5. The authors concluded that a higher Si/Al ratio resulted in a higher adhesion strength. Since geopolymer material with high Si/Al ratio represents a mixture of a partially reacted fly ash in sodium silicate, the medium may be the reason for the strong adhesion between coating and metal substrate.

Khan, et al. [13], studied the effect of Na/Al ratio and Si/Al ratio on the adhesion strength of fly ash-based geopolymer coatings. Mild steel was used as the substrate and a dip coating technique was applied to coat the substrate. Coated samples were cured at 60 °C for three days, seven days, and 28 days. A maximum adhesion strength of 10 MPa was obtained for a sample which was cured for 28 days indicating that a longer curing time will result in higher adhesion strength. In addition, an increase in Si/Al ratio and Na/Al ratio will result in a higher adhesion strength. Compared to geopolymer coating with a Si/Al ratio of 2.0, geopolymer coating with Si/Al ratio of 3.0 achieved higher adhesion strength with the substrate due to a higher dissolved silica content which formed a condensed structure and higher surface roughness. Geopolymer coating with Na/Al ratio of 0.8 achieved lower adhesion strength compared to a coating with Na/Al of 1.0. This is due to higher content of sodium hydroxide which is a key factor in the geopolymerization process.

Khan, et al. [14], expanded the above study by investigating the effect of Na/Al ratios and water to solid (W/S) ratios on the adhesion strength of a sodium silicate-free geopolymer coating. Fly ash was used as aluminosilicate source for making a geopolymer coating while steel plate served as substrate. The coating was applied using a dipping technique and the coated substrates were cured at 60 °C for 3, 7, 28, and 180 days. The maximum adhesion strength obtained was 3.8 MPa after curing for three days. There were no significant changes in adhesion strength when samples were cured longer than 28 days. A higher W/S ratio will result in lower adhesion strength caused by dilution of the activator. High adhesion strength can be achieved when the Na/Al ratio is between 0.8 and 1.2, W/S ratio of 0.33, and sample curing at 60 °C.

Despite the high number of publications on polymer coatings reported in the literature in recent years [15,16,17,18,19,20], most studies adopted the one-factor-a-time (OFAT) approach. A more efficient analytical tool, such as RSM (a mathematical model), is a prerequisite in order to predict the optimum composition of a GCC with a high adhesion strength. Compared to OFAT, design of experiments (DOE) provides many advantages, including low resource requirements (experimental runs, time, material and manpower), accurate measurement of main effects and interactions, and the ability to analyze several variables at the same time [21]. RSM, originally coined by Box and Wilson [22], is used commonly as a mathematical model for figuring out significant effects, interactions and optimization studies. The central composite design (CCD) has been proven to be the best model for analysis purposes [23]. Table 1 shows the difference in the total number of experimental runs between a full RSM design and a full factorial (classical method) design. The comparison is based on different number of factors (parameters) at 5-factor levels. For example, if an experiment compared the temperature of 10, 20, 30, 40, and 50 °C, then the factor “temperature” would have five levels which are 10, 20, 30, 40, and 50 °C. The result shows that for analyzing four factors, full design of RSM only requires at least 31 experimental runs (one replication) as compared to 625 for a full factorial design.

Since response surface methodology (RSM), specifically the CCD (a type of RSM design), has been widely used in polymer optimization [17,24,25,26], it will, therefore, be adopted in this study. The main objectives of this paper are: (i) to determine the significance of each of two factors (RHA/AA ratio and curing temperature) and their interaction on the adhesion strength of RHA-based GCC, (ii) to elucidate the relationship between the factors and the response under adhesion test, (iii) to optimize the factors to provide the maximum adhesion strength of GCC, and (iv) to elucidate the material behavior of GCC under similar tests.

## 2. Materials and Methods

### 2.1. Factors and Levels of the Design of Experiment (DOE)

In the study, RHA/AA ratio (*V*_1_) and curing temperature (*V*_2_) were chosen as factors. Based on the preliminary result via a screening process using a fractional factorial design (FrFD), other factors such as the ratio of AA solution, the NaOH concentration and curing time were kept constant at 5.5, 12 M and seven days, respectively. Factors and levels used in the DOE are shown in Table 2.

### 2.2. Design of Experiments

At each design stage, five levels and two factors were applied with a CCD and two replications for a total of 26 experimental runs. The factors were selected based on previous studies, their significant effect on the responses, and working range (workability). Table 3 displays the complete CCD with coded and uncoded levels of the factors. The value for the total block is 1 with the experiments carried out in randomized order.

The significance of the main factors and their interactions was calculated via an analysis of variance (ANOVA) using MINITAB @ 16.2 (Minitab, LLC, State College, PA, USA). The value of 95% was set as the significance level which reflected the *p*-value of 0.05. Based on the value of the correlation coefficient (R^2^), the regression coefficient model (mathematical model) developed in the ANOVA table was used for purposes of optimization. In order to acquire the regression coefficient model, experimental data were fitted with the second-order polynomial model. The general mathematical model obtained from the analysis is shown in Equation (1):

(1) $$ϒ=\beta_{0}+\sum_{t=1}^{3}\beta_{i}X_{i}+\sum_{i}^{3}\beta_{ii}X_{i}^{2}+\sum_{i-1}^{2}\sum_{j=i+1}^{3}\beta_{ij}X_{i}X_{j}$$

where ϒ is the response, *β*_0_, *β_i_*, *β_ii_*, and *β_ij_* are regression coefficients for the intercept, linear, quadratic, and interaction terms, respectively. *X_i_* and *X_j_* are coded values for the independent variables [27].

### 2.3. Raw Materials and Sample Preparation

Rice husk, which was used as the main component in the geopolymer production, was supplied by Maero Tech Sdn. Bhd (Nilai, Malaysia). The material was processed in a Pulverisette 4 planetary mill (FRITSCH GmbH—Milling and Sizing, Idar-Oberstein, Germany) to produce rice husk ash (RHA). The RHA was sieved and a particle size distribution (PSD) test for particles passing through an opening of 75 microns was conducted. The average particle size was 22.8 µm with 10% of the particles recorded as finer than 3.4 µm. Its specific surface area was 0.701 m^2^/g. The main components in RHA are shown in Table 4. Silicon dioxide (SiO_2_), aluminium oxide (Al_2_O_3_)_,_ and palladium(II) oxide (PdO) were found to be the major constituents, followed by iron(III) oxide (Fe_2_O_3_) and calcium oxide (CaO). Other elements, totaling 1.063% by mass, include potassium oxide (K_2_O), chromium(III) oxide (Cr_2_O_3_), manganese(II) oxide (MnO), nickel(II) oxide (NiO), and copper(II) oxide (CuO). Unknown elements which were burned or volatilized when heated at high temperature is reported as losses on ignition (LOI). 

Mild steel plate (50 mm × 50 mm × 1 mm) was scrubbed using sandpaper to remove any rust and to make the surface rougher, before cleaning using detergent, distilled water and acetone. The sodium hydroxide used was fixed at 12 molar. GCC samples were fabricated based on different RHA/AA ratios and curing temperatures as shown in Table 3.

The GCC was first prepared by mixing sodium hydroxide and sodium silicate solution at a designated ratio to form an AA solution which was then mixed with RHA. The mixture was mechanically stirred at low speed of approximately 100 rpm for 30 min. It was then coated on the mild steel substrate through a sieve and placed in a vacuum oven for 15 min to remove large and small bubbles. The coating was pressed using a hot press to form an average coating thickness of 1 ± 0.1 mm. The coated substrate was next placed in an oven for 24 h at ambient temperature for the next six days to complete the curing.

### 2.4. Microstructure Test

A S-3400N scanning electron microscope (SEM, Hitachi, Tokyo, Japan) was used to examine the microstructure of samples with the highest and lowest adhesion value. The samples were mounted on an 8 mm diameter stub with a carbon conductive tape and sputter-coated with gold in a vacuum JSM-IT100 (InTouchScope, Hitachi). All samples were examined at 1.0 kV for 100× and 1000× magnifications.

### 2.5. Adhesion Test

**Figure 1:**
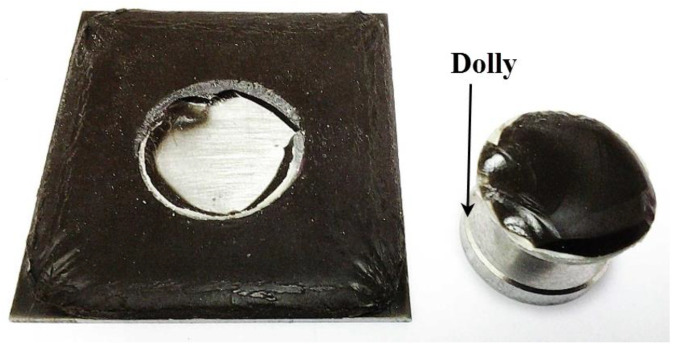
Sample that has undergone adhesive failure.

An elcometer 106 pull-off adhesion tester (0 MPa to 7 MPa) was used in accordance with the ASTM D4541 standard. Based on this method, we can measure the maximum tensile strength by estimating the perpendicular force per unit area immediately before a dolly detaches from a surface. The procedure was conducted by employing a pull-off adhesion test in determining the adhesion strength between substrate and coating. Figure 1 shows a coated sample which underwent adhesive failure.

**Figure 2:**
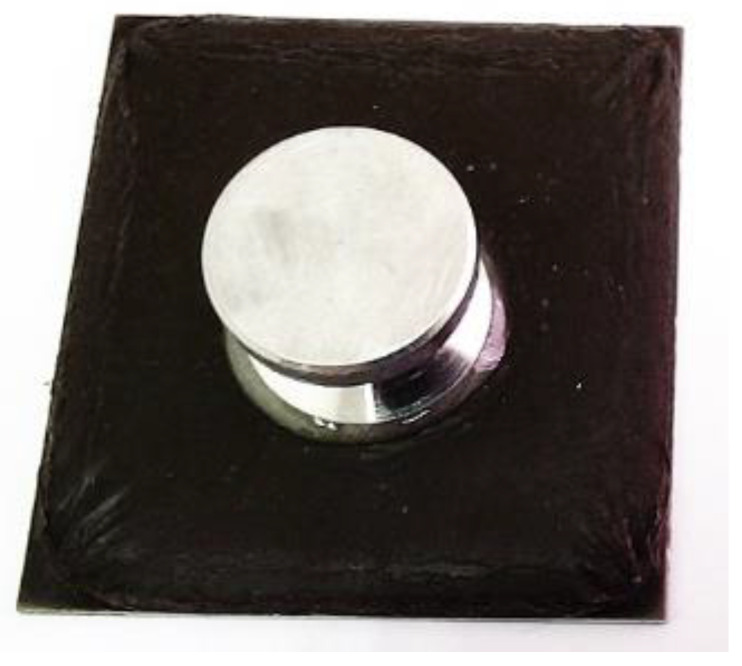
Dolly applied on the coating surface.

Prior to testing, the specimen was dried to ensure minimum content of moisture. The surface of the dolly was cleaned using sandpaper and acetone in order to remove oil and dirt. Araldite ‘rapid five minutes’ comprising epoxy resin and hardener, was mixed at 1:1 ratio and applied thinly on to the dolly. The dolly was pressed firmly on the coating surface for two minutes and allowed to cure in temperatures between 38 °C to 42 °C for at least 24 h as shown in Figure 2.

**Figure 3:**
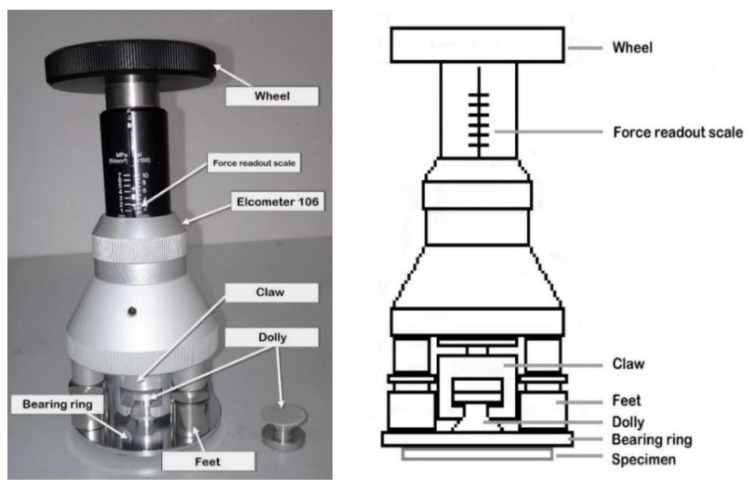
Elcometer 106 Pull Off Adhesion Tester.

Figure 3 shows a full set of elcometer 106 including wheel, force readout scale, claw, dolly, bearing ring and feet. During the test, the bearing ring was placed concentrically around the dolly and the force readout was set to 0 MPa by lowering the claw mechanism. The elcometer was then slid onto the specimen in such a way that all three feet rested on the bearing ring and the claw engaged with the dolly. The wheel was turned and in sequence it tightened the mechanism and raised the claw. The wheel was turned at a constant rate to ensure accurate reading. Once the coating was detached from the substrate, the dolly was pulled away from the surface emitting a loud ‘pop’ sound. The force required to pull the coating off the substrate was recorded by the force readout scale.

### 2.6. Microstructure of Rice Husk Ash (RHA)

**Figure 4:**
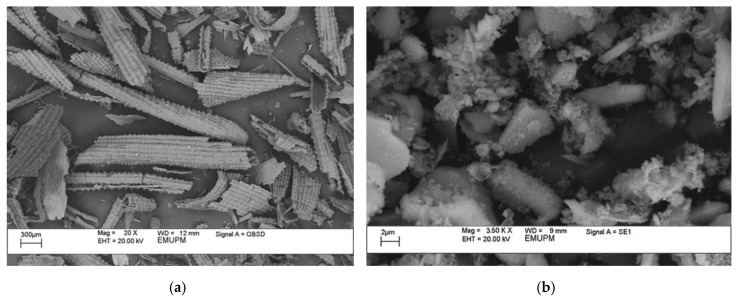
SEM images of Rice Husk Ash (RHA) particles (**a**) before and (**b**) after grinding.

Figure 4a shows the SEM images of burnt RHA prior to the grinding process. The size of RHA particles varied widely between 1 µm and 100 µm. Most of the particles acquired a platy, thin shell-like shape and the surface showed rectangular indents, a pattern probably inherited from the original structure of rice husk (RH). Due to this shape, RHA has a characteristic very high specific area. In addition, these sponge-like particles were irregular in shape and have porous cellular surfaces.

Image of the ground RHA was obtained using scanning electron microscopy (SEM) as shown in Figure 4b. RHA particles containing primarily silicon, with amorphous shapes as with cristobalite and crystalline quartz, were found to be solid in nature [28,29,30]. And when calcinated at a high temperature will produce crystalline RHA with amorphous shapes [31]. The uneven size of the particles was due to the high combustion temperature applied on the RH.

RHA particles have been observed to be much smaller after grinding and display a more homogenous distribution. The finer particle size during mixing and curing improved the reactivity [32,33]. In addition, due to the high surface energy and free OH groups on the silica surface, some particles can be seen as agglomerated and bound to each other. Hydrogen bonds produced with water molecules were also displaced by Si-O-Si bonds formed when dispersed silica were isolated from the solvent [34].

## 3. Results and Discussion

Data were analyzed using MINITAB software and the complete design matrix and response values of adhesion strength are given in Table 5.

### 3.1. Statistical Analysis of Adhesion Strength

One of the criteria for determining the best-fitted model was by considering the P which provides a significant effect in the model as seen in Table 6. The results indicated that both factors and interaction effects were significant at 95.00% confidence level. The *p* of regression analysis of all factors and their interactions were highly significant (*p* < 0.000) except for RHA/AA ratio with *p* < 0.004. Value for R^2^ = 0.9821 and R^2^ (adjusted) = 0.9752 were considered very high which indicated that 98.21% of the sample variation in the response was attributed to the factors.

Equation (2) represents the regression model for adhesion strength:

(2) $${ϒ}_{AS}=2.9733-0.1083\left(V_{2}\right)+0.2333\left(V_{3}\right)-0.2500\left(V_{2}{}^{2}\right)-0.2250\left(V_{3}{}^{2}\right)-0.8750\left(V_{2}V_{3}\right),$$

where ϒ*_AS_* represents the response (adhesion strength) and *V*_1_ and *V*_2_ are the decoded values of RHA/AA ratio and curing temperature respectively. This regression model can be used to calculate and analyze the effect of factors on adhesion strength of the RHA-based geopolymer coating.

### 3.2. Effect of Factors on Adhesion Strength

**Figure 5:**
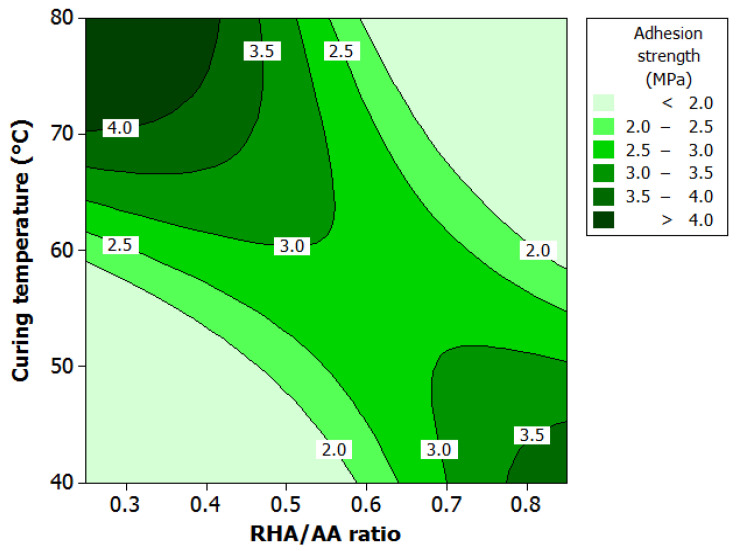
Contour plot for the effect of RHA/AA ratio and curing temperature on the adhesion strength.

ANOVA and regression model were used to analyze the effects of various factors on adhesion strength. Contour plots were used for better illustration. Figure 5 illustrates the effect of RHA/AA ratio (*V*_2_) and curing temperature (*V*_3_) on adhesion strength. High adhesion strength can be obtained under two situations; (i) higher *V*_3_ and a lower *V*_2_, or (ii) lower *V*_3_ and a higher *V*_2_. Although high adhesion strength can be obtained in this manner, the highest adhesion strength of greater than 4.0 MPa can only be achieved at much higher *V*_3_ and a lower *V*_2_. The increase in *V*_3_ indicates the extent to which the dissolution of precursors, mainly Al and Si, will be increased resulting in the consequent enhancement of the polycondensation process attributed to the increase in nucleation rates [35].

**Figure 6:**
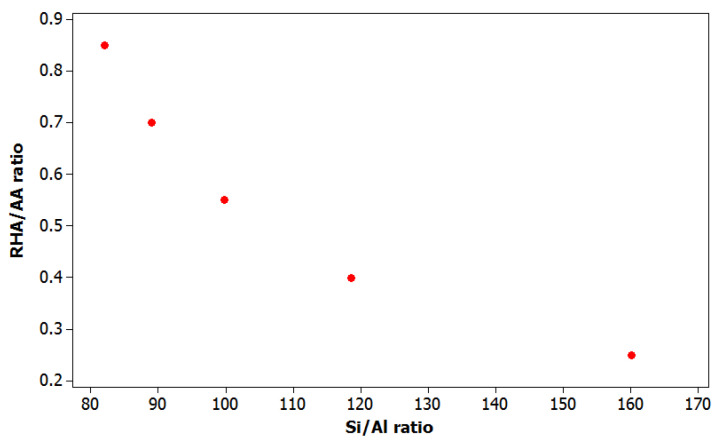
Scatterplot of RHA/AA ratio and Si/Al ratio for adhesion strength.

Since the lower *V*_2_ indicates higher Si/Al ratio as shown in Figure 6, the higher soluble silicate concentration in the solution accelerates the kinetic of the reactions thus enhancing geopolymerization. Higher *V*_3_ causes faster dissolution and higher Si/Al ratio effects faster synthetic geopolymeric gel growth as discussed previously by Yong, et al. [36]. The overall setting time, which is the time when geopolymer binder stops flowing will be much shorter.

**Figure 7:**
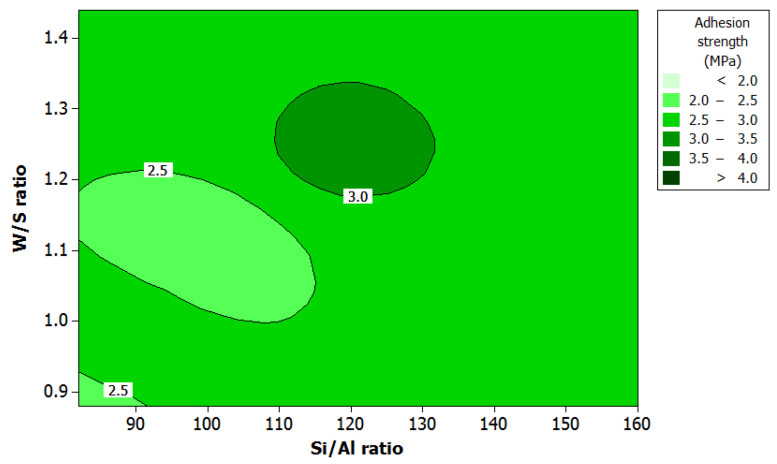
Contour plot of adhesion strength, W/S ratio, and Si/Al ratio.

Figure 7 shows the effect of W/S ratio and Si/Al ratio on adhesion strength. The optimum Si/Al ratio was between 110 and 130. A Si/Al ratio lower than 110 resulted in lower extent of dissolution and consequently reduction in rate of nucleation. This will lead to longer setting time compared to that under higher silicate ratio.

A Si/Al ratio exceeding than 130 indicates excess silicate concentration which will probably reduce reactivity by inhibiting further condensation thus delaying the setting time. The results are similar to those reported in the literature [11]. According to Khan, Azizli, Sufian and Man [14], the setting time is also affected by the W/S ratio. Water content has a vital function as a reactant in geopolymerization [37]. A decrease in W/S ratio will lower the workability of the geopolymer system resulting in a high viscosity system and leading to a lower setting time [38].

In addition, a higher water content will result in higher setting time and low adhesion strength due to dilution of the activator. The results in this study are in agreement with those reported by Khan et al. [14] and Temuujin et al. [10]. The optimum W/S ratio was between 1.2 and 1.3, which indicated a moderate setting time. Temuujin et al. [10], found that the large amount of weakly bound water may be one of the reasons for the strong adhesion of geopolymer composite coating to metal substrate.

### 3.3. Optimization of the Responses

**Figure 8:**
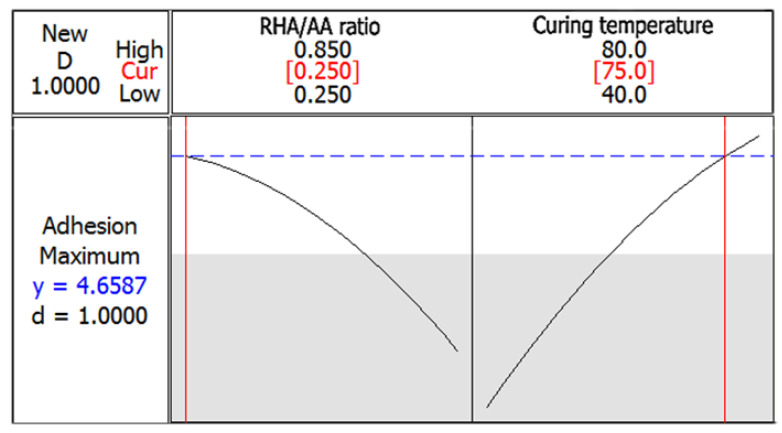
Optimization plot for adhesion strength.

Figure 8 shows the optimization plot which displays the combination of factors that produced the optimum predicted responses. Both the lower and target values were set at 1.8 and 4.4 MPa respectively. The maximum adhesion strength of 4.7 MPa can be achieved with the combination of RHA/AA ratio (*V*_2_) of 0.25 and curing temperature (*V*_3_) of 75 °C. The desirability of optimization was calculated as 1.0000 indicating that all parameters were within the target which was to obtain the maximum adhesion strength.

### 3.4. Experimental Validation

Table 7 shows that the average error for adhesion strength was well below this value at only 5.68% thus leading to the conclusion that the regression model established using this method was able to accurately optimize the value for adhesion strength.

### 3.5. Coating Adhesion Behavior and Microstructure Analysis

**Figure 9:**
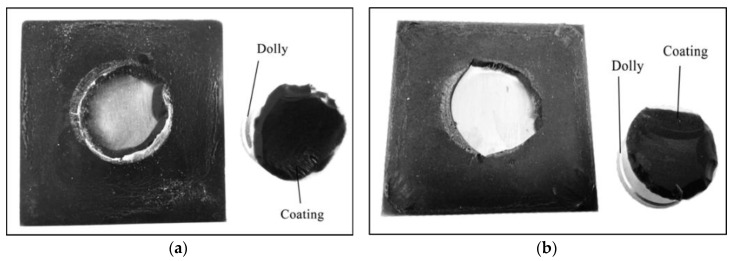
Adhesive failure on (**a**) sample S17 and (**b**) sample S26.

In this section, the coating samples which exhibited the highest and lowest adhesion strength were examined in order to determine the reason behind its contrasting performance. Sample S17 with adhesion strength of 4.4 MPa and S26 with adhesion strength of 1.8 MPa were chosen for this purpose. Following the adhesion test, both samples underwent adhesive failure which depicted by the area of the dolly, which was entirely covered by the coating as shown in Figure 9.

**Figure 10:**
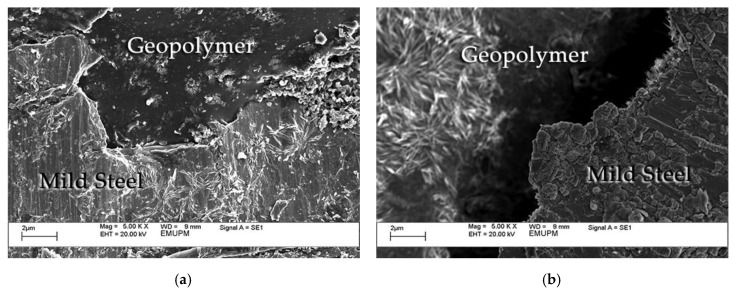
Cross-section SEM micrographs of (**a**) coated sample S17 and (**b**) coated sample S26.

SEM images were taken on cross-sections of coated samples to record the microstructure of the geopolymer binder on the mild steel substrate. Micrograph images as shown in Figure 10 were taken at the standard magnification of 500×. It was clearly shown that sample S17, which exhibited the best adhesion strength, had a good interface bonding between coating and substrate.

Conversely however, sample S26 showed very poor interface bonding since a gap was recorded between the coating and substrate. Factors that may influence adhesion performance of the coating material include substrate surface roughness, the coating composition, and type of interface bonding.

Surface roughening is a means by which the mechanical bonding may be enhanced by facilitating interlocking. According to Temuujin et al. [11], substrate surface roughness is a factor that influences the adhesion strength. Figure 10a shows that the coating penetrated the valleys of the profiles, wetting the entire substrate surface thus providing excellent adhesion properties. Conversely, poor adhesion bonding is produced where the coating failed to penetrate, as seen in Figure 10b.

**Figure 11:**
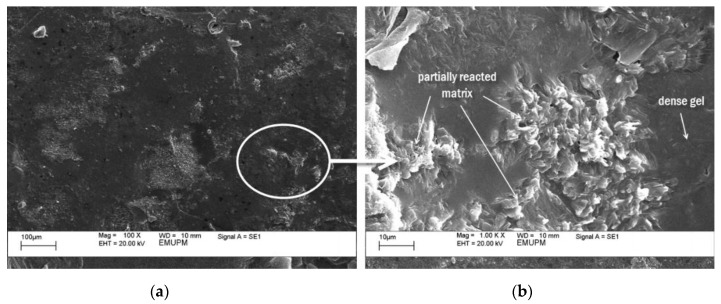
SEM micrograph of geopolymer binder with the highest adhesion strength (sample S17). (**b**) is the magnification of (**a**).

**Figure 12:**
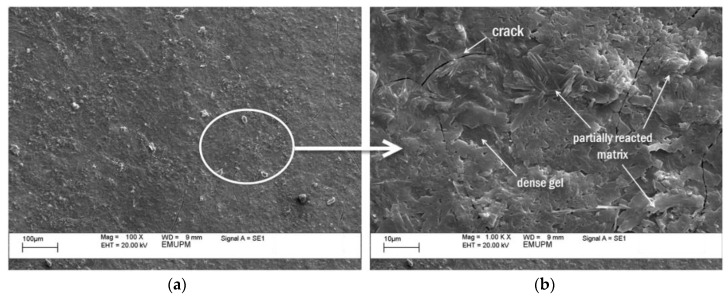
SEM micrograph of geopolymer binder with the lowest adhesion strength (sample S26). (**b**) is the magnification of (**a**).

Figure 11a and Figure 12a show SEM micrographs of geopolymer binder for both samples. Figure 11b and Figure 12b are the magnification of Figure 11a and Figure 12a, respectively. Both samples contained partially reacted matrix and dense gel. The difference between them is the portion of the activated raw material and existence of cracks. As can be seen in Figure 11, there is a large portion of the activated raw materials which transformed into a dense gel matrix. This dense gel phase has a well-connected structure consisting of the glassy phase. A previous study had noted that an increase in Si/Al ratio will lead to higher degree of dissolution, gel formation, and denser gel structure [39]. The coating completely wetted the surface of the substrate. And given the large contact surface area between gel matrix and substrate surface, excellent adhesion bonding was produced.

Figure 12 shows the geopolymer surface covered with cracks. Formation of cracks weakened the geopolymer structure leading to poor adhesion strength. A large force is required to separate the strong adhesion of the interface while a smaller force exerted on the weak adhesion interface is enough to make the crack propagate [40]. Sample S26 which had lower water to solid (W/S) ratio of 1.10, compared to 1.24 for sample S17, was less elastic and viscous. The coating tended to separate easily from the substrate since it was unable to deform and it also does not have any internal mechanisms to dissipate energy. Therefore, less energy is needed to counter the breakdown in interface strength as discussed previously by Grillet et al. [41]. In addition, the geopolymer matrix in sample S26 was covered with the partially reacted matrix. Only small parts of this comprised of dense gel. When the geopolymer binder was applied on the substrate, the gel matrix reacted to form a highly crystalline geopolymer structure as shown in Figure 10b which may contribute to poor interface bonding. He et al. [42] determined that a large quantity of unreacted or partially reacted particles will result in some negative effects on the strength of the end products.

## 4. Conclusions

Response surface methodology (RSM) proved to be an adequate tool to study the relationship between factors which significantly influenced adhesion properties. From the analysis and investigation, adhesion strength of geopolymer composite coating (GCC) was highly significantly influenced by all factors and their interactions (*p* < 0.000) except for RHA/AA ratio which was very significant at *p* < 0.004. A maximum adhesion strength of 4.7 MPa for GCC was achieved with the combination of RHA/AA ratio and curing temperature of 0.25 and 75 °C, respectively. The optimum adhesion strength for GCC, greater than 3.0 MPa, was achieved with the Si/Al ratio within the range 110 to 130 and the W/S ratio within 1.2 to 1.3. GCC with low and high adhesion strengths of 1.8 MPa and 4.4 MPa, respectively, displayed adhesive failure in their coatings. Coating with good interfacial bonding with the substrate had good wetting ability in which the coating penetrated the valley of the profiles thus wetting the entire substrate surface. A large portion of dense gel matrix also contributed to high adhesion strength. Conversely, large quantities of unreacted or partially reacted particles may result in low adhesion strength. Several potential studies have been identified including the effect of palladium (II) oxide on the geopolymer properties.

## Figures and Tables

**Table 1:** Total number of experimental runs for full factorial design and RSM based on 5-level factors.

Number of Factors	Levels	Total Number of Experimental Runs	
		Full Factorial Design	RSM
4	5	625	31
5	5	3125	54
6	5	15,625	90
7	5	78,125	160

**Table 2:** Factors and levels used for adhesion test.

Factor	Unit	Notation	Levels				
			−2	−1	0	1	2
RHA/AA ratio	-	*V*_1_	0.25	0.40	0.55	0.70	0.85
Curing temperature	°C	*V*_2_	40	50	60	70	80

**Table 3:** Design matrix.

Sample	Coded Factor		Uncoded Factor	
	*V*_1_	*V*_2_	*V*_1_	*V*_2_
S1	0	0	0.55	60
S2	2	0	0.85	60
S3	0	0	0.55	60
S4	0	0	0.55	60
S5	−1	1	0.40	70
S6	−1	−1	0.40	50
S7	0	2	0.55	80
S8	1	−1	0.70	50
S9	−2	0	0.25	60
S10	1	1	0.70	70
S11	0	0	0.55	60
S12	0	0	0.55	60
S13	0	−2	0.55	40
S14	0	−2	0.55	40
S15	−1	−1	0.40	50
S16	1	1	0.70	70
S17	−1	1	0.40	70
S18	0	2	0.55	80
S19	0	0	0.55	60
S20	0	0	0.55	60
S21	0	0	0.55	60
S22	2	0	0.85	60
S23	0	0	0.55	60
S24	−2	0	0.25	60
S25	1	−1	0.70	50
S26	0	0	0.55	60

**Table 4:** Composition in RHA.

Element	SiO_2_	PdO	Al_2_O_3_	Fe_2_O_3_	CaO	Others	LOI
Mass%	87.4	5.0	3.0	1.49	1.40	1.063	0.647

**Table 5:** Design matrix and response value for the adhesion test.

Sample	RHA/AA Ratio (*V*_1_)	Curing Temperature (*V*_2_)	RHA/AA Ratio (*V*_1_)	Curing Temperature (*V*_2_)	Adhesion Strength (MPa)
S1	0	0	0.55	60	2.0
S2	2	0	0.85	60	2.4
S3	0	0	0.55	60	2.1
S4	0	0	0.55	60	1.9
S5	−1	1	0.40	70	4.2
S6	−1	−1	0.40	50	2.1
S7	0	2	0.55	80	3.0
S8	1	−1	0.70	50	3.4
S9	−2	0	0.25	60	2.7
S10	1	1	0.70	70	2.2
S11	0	0	0.55	60	2.1
S12	0	0	0.55	60	2.0
S13	0	−2	0.55	40	2.2
S14	0	−2	0.55	40	2.2
S15	−1	−1	0.40	50	2.0
S16	1	1	0.70	70	2.3
S17	−1	1	0.40	70	4.4
S18	0	2	0.55	80	3.0
S19	0	0	0.55	60	1.8
S20	0	0	0.55	60	1.9
S21	0	0	0.55	60	1.8
S22	2	0	0.85	60	2.3
S23	0	0	0.55	60	1.8
S24	−2	0	0.25	60	2.6
S25	1	−1	0.70	50	3.6
S26	0	0	0.55	60	1.8

**Table 6:** Estimated effects and coefficient for adhesion strength.

Term	Notation	Coefficient	Std. Error of Coefficient	*p*
Constant		2.9733	0.06176	0.000
Block 1		0.5017	0.07756	0.000
Block 2		−1.0533	0.06523	0.000
RHA/AA ratio	*V*_1_	−0.1083	0.03317	0.004
Curing temperature	*V*_2_	0.2333	0.05245	0.000
RHA/AA ratio ∗ RHA/AA ratio	*V*_1_ ∗ *V*_1_	−0.2500	0.03047	0.000
Curing temperature ∗ Curing temperature RHA/AA ratio ∗ Curing Temperature	*V*_2_ ∗ *V*_2_ *V*_1_ ∗ *V*_2_	−0.2250 −0.8750	0.03047 0.04063	0.000 0.000

R^2^ = 0.9821; R^2^ (adj) = 0.9752.

**Table 7:** Experimental validation for adhesion strength.

Sample	Adhesion Strength (MPa)		
	Experimental Value	Predicted Value	Error (%)
SV1	4.6	4.7	2.13
SV2	4.8	4.7	2.13
SV3	4.1	4.7	12.77
	$\bar{x}$ Error		5.68

## References

[1] Padture N.P., Gell M., Jordan E.H. (2002). Thermal barrier coatings for gas-turbine engine applications. Science.

[2] Hull T., Colligon J., Hill A. (1987). Measurement of thin film adhesion. Vacuum.

[3] Temuujin J., Minjigmaa A., Rickard W., van Riessen A. (2012). Thermal properties of spray-coated geopolymer-type compositions. J. Therm. Anal. Calorim..

[4] Mohd Basri M.S., Mustapha F., Mazlan N., Ishak M.R. (2016). Fire retardant performance of rice husk ash-based geopolymer coated mild steel-A factorial design and microstructure analysis. Proceedings of Materials Science Forum.

[5] Basri M.S.M., Mustapha F., Mazlan N., Ishak M.R. (2020). Optimization of rice husk ash-based geopolymers coating composite for enhancement in flexural properties and microstructure using response surface methodology. Coatings.

[6] Xie J., Chen W., Wang J., Fang C., Zhang B., Liu F. (2019). Coupling effects of recycled aggregate and GGBS/metakaolin on physicochemical properties of geopolymer concrete. Constr. Build. Mater..

[7] Messina F., Ferone C., Molino A., Roviello G., Colangelo F., Molino B., Cioffi R. (2017). Synergistic recycling of calcined clayey sediments and water potabilization sludge as geopolymer precursors: Upscaling from binders to precast paving cement-free bricks. Constr. Build. Mater..

[8] Colangelo F., Roviello G., Ricciotti L., Ferrandiz-Mas V., Messina F., Ferone C., Tarallo O., Cioffi R., Cheeseman C.R. (2018). Mechanical and thermal properties of lightweight geopolymer composites. Cem. Concr. Compos..

[9] Davidovits J. (1993). From ancient concrete to geopolymers. Art Metiers Mag.

[10] Temuujin J., Minjigmaa A., Rickard W., Lee M., Williams I., Van Riessen A. (2009). Preparation of metakaolin based geopolymer coatings on metal substrates as thermal barriers. Appl. Clay Sci..

[11] Temuujin J., Minjigmaa A., Rickard W., Lee M., Williams I., Van Riessen A. (2010). Fly ash based geopolymer thin coatings on metal substrates and its thermal evaluation. J. Hazard. Mater..

[12] Yusof A.M., Nizam N.A., Abd Rashid N.A. (2010). Hydrothermal conversion of rice husk ash to faujasite-types and NaA-type of zeolites. J. Porous Mater..

[13] Khan M.I., Azizli K., Sufian S., Man Z. (2014). Effect of Na/AI and Si/AI ratios on adhesion strength of geopolymers as coating material. Appl. Mech. Mater..

[14] Khan M.I., Azizli K., Sufian S., Man Z. (2015). Sodium silicate-free geopolymers as coating materials: Effects of Na/Al and water/solid ratios on adhesion strength. Ceram. Int..

[15] Sulaiman A., Silva F.V. (2013). High pressure processing, thermal processing and freezing of ‘Camarosa’strawberry for the inactivation of polyphenoloxidase and control of browning. Food Control.

[16] Sulaiman A., Soo M.J., Yoon M.M., Farid M., Silva F.V. (2015). Modeling the polyphenoloxidase inactivation kinetics in pear, apple and strawberry purees after high pressure processing. J. Food Eng..

[17] Azeem B., KuShaari K., Naqvi M., Kok Keong L., Almesfer M.K., Al-Qodah Z., Naqvi S.R., Elboughdiri N. (2020). Production and characterization of controlled release urea using biopolymer and geopolymer as coating materials. Polymers.

[18] Lian Y.-S., Sun J.-Y., Zhao Z.-H., Li S.-Z., Zheng Z.-L. (2020). A refined theory for characterizing adhesion of elastic coatings on rigid substrates based on pressurized blister test methods: Closed-form solution and energy release rate. Polymers.

[19] Sherif G., Chukov D.I., Tcherdyntsev V.V., Torokhov V.G., Zherebtsov D.D. (2020). Effect of glass fibers thermal treatment on the mechanical and thermal behavior of polysulfone based composites. Polymers.

[20] Cheng L., Ren S., Lu X. (2020). Application of eco-friendly waterborne polyurethane composite coating incorporated with nano cellulose crystalline and silver nano particles on wood antibacterial board. Polymers.

[21] Czitrom V. (1999). One-factor-at-a-time versus designed experiments. Am. Stat..

[22] Box G.E., Wilson K.B. (1992). On the experimental attainment of optimum conditions. Breakthroughs in Statistics.

[23] Czyrski A., Jarzębski H. (2020). Response surface methodology as a useful tool for evaluation of the recovery of the fluoroquinolones from plasma—The study on applicability of box-behnken design, central composite design and doehlert design. Processes.

[24] Flaifel M.H. (2020). An approach towards optimization appraisal of thermal conductivity of magnetic thermoplastic elastomeric nanocomposites using response surface methodology. Polymers.

[25] Hassan M.Z., Roslan S.A., Sapuan S., Rasid Z.A., Mohd Nor A.F., Md Daud M.Y., Dolah R., Mohamed Yusoff M.Z. (2020). Mercerization optimization of bamboo (bambusa vulgaris) fiber-reinforced epoxy composite structures using a box–behnken design. Polymers.

[26] Chen Y., Wang F., Dong L., Li Z., Chen L., He X., Gong J., Zhang J., Li Q. (2019). Design and optimization of flexible polypyrrole/bacterial cellulose conductive nanocomposites using response surface methodology. Polymers.

[27] Tabaraki R., Nateghi A. (2011). Optimization of ultrasonic-assisted extraction of natural antioxidants from rice bran using response surface methodology. Ultrason. Sonochemistry.

[28] Shinohara Y., Kohyama N. (2004). Quantitative analysis of tridymite and cristobalite crystallized in rice husk ash by heating. Ind. Health.

[29] Chaudhary D.S., Jollands M.C. (2004). Characterization of rice hull ash. J. Appl. Polym. Sci..

[30] Chaudhary D.S., Jollands M.C., Cser F. (2004). Recycling rice hull ash: A filler material for polymeric composites?. Adv. Polym. Technol..

[31] Xu H., Van Deventer J.S. (2003). Effect of source materials on geopolymerization. Ind. Eng. Chem. Res..

[32] Hardjito D., Rangan V. (2005). Development and Properties of Low-Calcium Fly Ash-Based Geopolymer Concrete.

[33] Chindaprasirt P., Chotetanorm C., Rukzon S. (2010). Use of palm oil fuel ash to improve chloride and corrosion resistance of high-strength and high-workability concrete. J. Mater. Civ. Eng..

[34] Thuc C.N.H., Thuc H.H. (2013). Synthesis of silica nanoparticles from Vietnamese rice husk by sol–gel method. Nanoscale Res. Lett..

[35] Sindhunata J.S.J., Lukey G.C., Xu H. (2006). Effect of curing temperature and silicate concentration on fly-ash-based geopolymerization. Ind. Eng. Chem. Res..

[36] Yong S., Feng D., Lukey G., Van Deventer J. (2007). Chemical characterisation of the steel–geopolymeric gel interface. Colloids Surf. A Physicochem. Eng. Asp..

[37] Rees C.A., Provis J.L., Lukey G.C., Van Deventer J.S. (2007). In situ ATR-FTIR study of the early stages of fly ash geopolymer gel formation. Langmuir.

[38] Nath P., Sarker P.K., Rangan V.B. (2015). Early age properties of low-calcium fly ash geopolymer concrete suitable for ambient curing. Procedia Eng..

[39] Rodriguez E.D., Bernal S.A., Provis J.L., Gehman J.D., Monzó J.M., Payá J., Borrachero M.V. (2013). Geopolymers based on spent catalyst residue from a fluid catalytic cracking (FCC) process. Fuel.

[40] Gong Y., Li S., Liu J. (2018). Peeling of an Extensible Soft Microbeam to Solid: Large Deformation Analysis. Int. J. Appl. Mech..

[41] Grillet A.M., Wyatt N.B., Gloe L.M. (2012). Polymer gel rheology and adhesion. Rheology.

[42] He J., Jie Y., Zhang J., Yu Y., Zhang G. (2013). Synthesis and characterization of red mud and rice husk ash-based geopolymer composites. Cem. Concr. Compos..

